# Community-based participatory research initiative to assess the scope of alcohol use within the community of refugees from Burma in the greater Denver area

**DOI:** 10.1192/j.eurpsy.2023.1730

**Published:** 2023-07-19

**Authors:** A. Franks, N. Arlas, M. Harrison, L. Officer, B. Fuller, D. Le, D. Hunt

**Affiliations:** 1 Psychiatry; 2 University of Colorado, Denver, United States

## Abstract

**Introduction:**

For over fifty years, minorities in Burma have faced severe persecution and violence, forcing them to flee their homeland. In the past ten years there has been an influx in the number of refugees resettled in Denver, Colorado. Refugees often struggle to navigate the complexities of the American health care system and adapt to life in a foreign culture. The development of programs and partnerships to assist refugees in their pursuit of health and integration is essential to building stronger communities.

**Objectives:**

This community based participatory research (CBPR) project was developed in collaboration with the refugee community from Burma living in the Denver area. After regular meetings with a group of motivated teenagers and young adults from this community to form our Youth Advisory Board (YAB), they identified alcohol use and misuse as a health concern within their community. With this identified issue, the project aimed to gather data from community members that could be leveraged to create, implement, and evaluate a culturally competent intervention to effectively address risky alcohol use in this community.

**Methods:**

Data collection involved formal one-on-one, semi-structured, audio-recorded interviews with community members. Participants were recruited voluntarily at health information nights held by the student researchers at their local apartment complex. The interviews were conducted by one medical student researcher with one translator present and were transcribed afterward. The interview data was analyzed using Immersion Crystallization methodology.

**Results:**

Initial results from the community meetings with the YAB, local organizations, formative community surveys, and key informant interviews highlighted the vulnerability of the refugee population, scarcity of culturally appropriate resources for alcohol abuse, and urgency of addressing problematic alcohol use. The analysis of the ten audio-recorded surveys showed several themes including negative consequences of alcohol use, specifically negative impacts on familial relationships, employment, and financial resources, and a perceived personal responsibility for managing one’s own alcohol consumption.

**Conclusions:**

This project corroborates current literature regarding the scope and breadth of hazardous alcohol use within the community of refugees from Burma. Our data has expanded our understanding of the values of community members including the influence of religion and family on behaviors, and the negative impact on employment as the most impactful negative consequence. These findings need to be shared with the community to move forward in mapping the most effective and appropriate interventions.

**Disclosure of Interest:**

None Declared

